# Dose-Dependent Analysis of Image Quality in Pediatric Head CT Scans Across Different Scanners to Optimize Clinical Protocols Using Phantom-Based Assessment

**DOI:** 10.3390/tomography11110119

**Published:** 2025-10-27

**Authors:** Hiroshi Kuwahara, Mitsuaki Ojima, Tsuneko Kawamura, Daisuke Saitou, Kazunari Andou, Eiji Ariga, Kotaro Hasegawa, Michiaki Kai

**Affiliations:** 1 Doctoral Course of Health Science, Graduate School of Nursing, Oita University of Nursing and Health Sciences, 2944-9 Megusuno, Oita City 870-1201, Oita Prefecture, Japan; 2 Department of Environmental Health Science, Oita University of Nursing and Health Sciences, 2944-9 Megusuno, Oita City 870-1201, Oita Prefecture, Japan; 3 Radiology Department, Nakatsu Municipal Hospital, 173-1 Shimoikenaga, Nakatsu City 871-8511, Oita Prefecture, Japan; radonsc@nakatsu-hosp.jp; 4 Radiology Department, Seichokai Social Medical Corporation Belland General Hospital, 500-3 Higashiyama, Naka Ward, Sakai City 599-8247, Osaka Prefecture, Japan; d_saito@seichokai.or.jp; 5 Department of Radiology, Oita Children’s Hospital, Fujimoto Ikuseikai Social Medical Corporation, 83-7 Katajima, Oita City 870-0943, Oita Prefecture, Japan; k.ando@oita-kodomo.jp; 6 Department of Radiology, Medical Technology Division, Nagoya Daini Red Cross Hospital, Japanese Red Cross Aichi Medical Center, 2-9 Myoken-cho, Showa-ku, Nagoya City 466-8650, Aichi Prefecture, Japan; ariga@nagoya2.jrc.or.jp; 7 Department of Radiologic Technology, Kariya Toyota General Hospital, Toyota Medical Corporation, 5-15 Sumiyoshi-cho, Kariya City 448-8505, Aichi Prefecture, Japan; kotaro.hasegawa@toyota-kai.or.jp; 8 Department of Health Science, School of Health Science, Nippon Bunri University, 1727 Ichiki, Oita City 870-0397, Oita Prefecture, Japan; kaima@nbu.ac.jp

**Keywords:** pediatric head CT, radiation dose, Catphan phantom, automatic exposure control, deep learning image reconstruction

## Abstract

**Simple Summary:**

Recent epidemiological research findings call for optimization of pediatric head computed tomography. Our multicenter study investigated how radiation dose influences image quality in pediatric head CT using both clinical data and Catphan phantom analysis across different CT systems. The results demonstrated that image noise decreased as dose increased, while contrast-to-noise ratio improved and reached a plateau above approximately 40 mGy. CT values were consistent among facilities, and no significant difference in figure of merit was observed across manufacturers. These findings indicate that acceptable image quality can be achieved at lower doses, supporting further protocol refinement to reduce radiation exposure without compromising diagnostic accuracy.

**Abstract:**

Background/Objectives: Optimization of pediatric head computed tomography (CT) protocols is essential to minimize radiation exposure while maintaining diagnostic image quality. Previous studies mainly relied on phantom-based measurements or visual assessments, and validation using clinical images remains limited. This study aimed to establish quantitative thresholds for noise and contrast-to-noise ratio (CNR) in pediatric head CT by integrating multicenter clinical data with phantom evaluations. Methods: A multicenter retrospective study was conducted using CT systems from eight hospitals, combined with Catphan phantom experiments and pediatric head CT data. Scan parameters, automatic exposure control settings, and reconstruction methods were collected. Image quality was quantified by the standard deviation (SD) of noise and CNR obtained from regions of interest in gray and white matter. Radiation dose was represented by CTDIvol. Relationships among CTDIvol, SD, and CNR were analyzed across scanners from three manufacturers (Canon, FUJI, and GE). Results: Consistent dose–response trends were observed across institutions and manufacturers. Image noise decreased as CTDIvol increased, but reached a plateau at higher doses. CNR improved with dose escalation, then stabilized. Both phantom experiments and clinical analyses identified a target SD of 5 and CNR of 2 as optimal indicators for pediatric head CT. Conclusions: Quantitative thresholds were determined as practical indicators for balancing diagnostic image quality with dose reduction. Further reduction may be achieved through advanced reconstruction methods, such as deep learning-based algorithms. These findings may contribute to standardizing pediatric head CT protocols and supporting safer and more effective diagnostic imaging.

## 1. Introduction

Traumatic brain injury (TBI) is a leading cause of death and disability among children over one year old, mainly resulting from motor vehicle accidents, falls, and abuse [1,2]. Advances in computed tomography (CT) technology have made it indispensable in emergency medicine due to its rapid acquisition, especially for detecting intracerebral and subarachnoid hemorrhages [2]. However, the rapid increase in CT examinations brought about concern about risk from CT radiation exposure [3]. The latest epidemiological studies, such as the EPI-CT study [4,5], have identified a small but significant increase in the risks associated with pediatric head CT scans, emphasizing the critical need to justify and optimize radiation exposure aligned with the International Commission on Radiological Protection’s recommendations [6,7,8]. Recent domestic research has also shown that the frequency of pediatric head CT examinations is influenced by clinical conditions such as hydrocephalus and congenital anomalies, underscoring the importance of justification in addition to optimization [9]. The EPI-CT study showed there were associations between brain dose from CT examinations and the risk of benign and malignant brain tumors. The Excess Relative Risk (ERR) per 100 mGy of all brain cancer was 1.27 (95% Confidence interval 0.51 to 2.69) [4], and all hematological malignancies had an ERR of 1.96 (95% confidence interval 1.10 to 3.12) per 100 mGy [5]. Mean cumulative brain dose was 47.4 mGy.

These findings underscore the importance of minimizing radiation exposure while maintaining diagnostic image quality, in accordance with the recommendations of the International Commission on Radiological Protection (ICRP) [6].

Radiation exposure in CT examinations is commonly quantified using standardized dose indices such as the volume CT dose index (CTDIvol), which reflects the scanner output for a specific imaging protocol and enables comparison of dose levels across different scanners and institutions [8]. Image quality, on the other hand, can be objectively assessed using physical parameters, including the standard deviation (SD) of pixel noise and the contrast-to-noise ratio (CNR), representing noise magnitude and lesion detectability. Previous pediatric head CT studies have used SD and CNR as key indicators to evaluate the relationship between radiation dose and diagnostic performance, demonstrating that maintaining appropriate noise levels and gray–white matter contrast ensures clinically acceptable image quality for hemorrhage detection [10,11,12]. While subjective evaluation by radiologists remains important, quantitative measures such as SD and CNR are essential for dose-optimization research because they enable reproducible, vendor-independent assessment of image performance. Therefore, evaluating both radiation dose and image-quality metrics in a standardized and quantitative manner is fundamental for establishing optimal CT protocols, particularly in pediatric populations where radiation risk and diagnostic reliability must be carefully balanced [8].

Whereas the introduction of clinical decision rules like PECARN, CATCH, and CHALICE [13] has reduced unnecessary CT scans [14], there is no unified international guideline for TBI imaging. In Japan, significant inter-facility variations in brain CT doses have been reported [15]. Diagnostic reference levels (DRLs) for pediatric CT have been established [16], underscoring the need to optimize doses further. Previous studies [17] used Catphan^®^ 600 and age-specific anthropomorphic phantoms to examine dose–image quality relationships. Although these phantom-based approaches provided valuable insights, they did not directly address how protocols should be optimized in real pediatric examinations. In recent years, iterative and deep learning-based reconstruction algorithms have further complicated the relationship between radiation dose and perceived image quality. Nevertheless, their clinical validation in pediatric cohorts remains limited. Therefore, this multicenter study aimed to optimize pediatric head CT imaging protocols based on two complementary analyses:

(1) Quantitative characterization of the dose–image-quality relationship across different CT systems using a Catphan phantom, and
(2) Validation of these relationships using actual pediatric head CT data collected from eight hospitals.

The primary optimization target was to identify dose-dependent thresholds of diagnostic image quality, balancing radiation exposure and lesion detectability in pediatric head CT. Image quality was assessed using SD and CNR as key metrics, and the combined results were used to identify dose-dependent thresholds for optimizing image quality in pediatric head CT protocols, particularly for hemorrhage screening in TBI.

## 2. Materials and Methods

### 2.1. Study Design and Ethics

This multicenter, retrospective study was conducted at eight medical facilities in Japan from February 2021 to March 2023. Pediatric head CT data from children aged 1–6 years were collected to assess image quality and radiation dose. Ethical approval was granted by the Institutional Review Board of Oita University of Nursing and Health Sciences (approval no. 22-73). The requirement for informed consent was waived due to the study’s retrospective nature and the use of anonymized data.

This study was designed to evaluate and optimize pediatric head CT protocols using both clinical data and phantom measurements under real-world conditions. All participating institutions followed identical data collection procedures to ensure inter-facility consistency.

### 2.2. Study Population and Data Collection

After excluding 13 cases with severe motion artifacts or missing metadata, 90 pediatric head CT cases were included. These patients underwent non-contrast CT due to suspected traumatic brain injury. All included cases were consecutive routine pediatric head CT examinations performed between February 2021 and March 2023. Because demographic information such as patient weight was anonymized across institutions, stratified analysis by body weight could not be performed. However, only children aged 1–6 years were included to minimize AEC variability related to body size. All scans were performed under routine clinical conditions with automatic exposure control (AEC). Acquisition parameters, such as tube voltage, tube current limits, scan time, pitch, and reconstruction methods, were recorded for each examination. Table 1 summarizes the CT systems used at each participating facility, whereas Table 2 details the scan conditions for pediatric head CT imaging at each facility. Only cases in which no intracranial hemorrhage was confirmed on diagnostic interpretation were included for image-quality analysis to avoid bias from pathological findings.

Images showing motion-related artifacts were also excluded to ensure consistent image quality assessment. As suggested by the reviewers, additional details regarding patient selection and exclusion criteria have been clarified to enhance methodological transparency. In consideration of patient privacy and institutional ethical standards, only age (1–6 years) was recorded as demographic information, while sex, height, and body weight were not collected. This policy was adopted to avoid potential identification of individual patients in a multicenter dataset. No additional exclusion criteria were applied.

### 2.3. CT Systems and Phantom Experiments

The CT scanners used in this study included Aquilion series systems (Canon Medical Systems Corporation, Otawara, Japan), Supria and SCENARIA series systems (Fujifilm Healthcare Corporation, Tokyo, Japan), and Revolution EVO (GE Healthcare Japan Corporation, Tokyo, Japan). All scanners were equipped with automatic exposure control (AEC) systems that allowed the target SD setting to be adjusted between 1 and 10. Image reconstruction was performed using either filtered backprojection (FBP) or hybrid iterative reconstruction (HIR, Smooth level 3). The presence or absence of HIR was recorded for each facility, as listed in Table 2b. Because each facility applied the reconstruction method routinely used in its clinical workflow, separate analyses for datasets with and without HIR were not performed. Instead, the reconstruction type (FBP or HIR) was recorded in Table 2b to reflect real-world practice. The potential influence of reconstruction method differences on image noise and CNR was discussed as a study limitation.

Quantitative image-quality evaluation was performed using the Catphan^®^ 500 phantom (CTP515 low-contrast module; The Phantom Laboratory, Salem, NY, USA).

The module design and specifications were based on the manufacturer’s technical data sheet [18]. The Catphan^®^ 500 phantom is made of water-equivalent epoxy resin with low-contrast inserts of varying densities (0.3%, 0.5%, and 1.0%) to simulate soft-tissue attenuation properties, although it does not reproduce the bioelectrical characteristics of human tissues. This material composition allows quantitative evaluation of image noise and contrast behavior under controlled and repeatable conditions. The CTP515 module has a diameter of 15 cm and a thickness of 40 mm. It consists of a water-equivalent epoxy resin background containing multiple cylindrical low-contrast rods arranged across three contrast levels (0.3%, 0.5%, and 1.0%) and varying diameters (2–15 mm). Both the background and low-contrast rods have equivalent effective atomic numbers, with only density differences used to achieve the desired contrast levels. The 40 mm rod length provides consistent contrast along the z-axis, minimizing partial-volume effects during helical scanning. Subslice targets (truncated cylinders) are also included to evaluate the effectiveness of reconstruction parameters in detecting small, low-contrast objects.

Images were evaluated in accordance with the Japanese Standard X-ray Computed Tomography (CT Image Measurement Method [19]), focusing on image noise (SD) and mean CT values in homogeneous regions.

**Figure 1:**
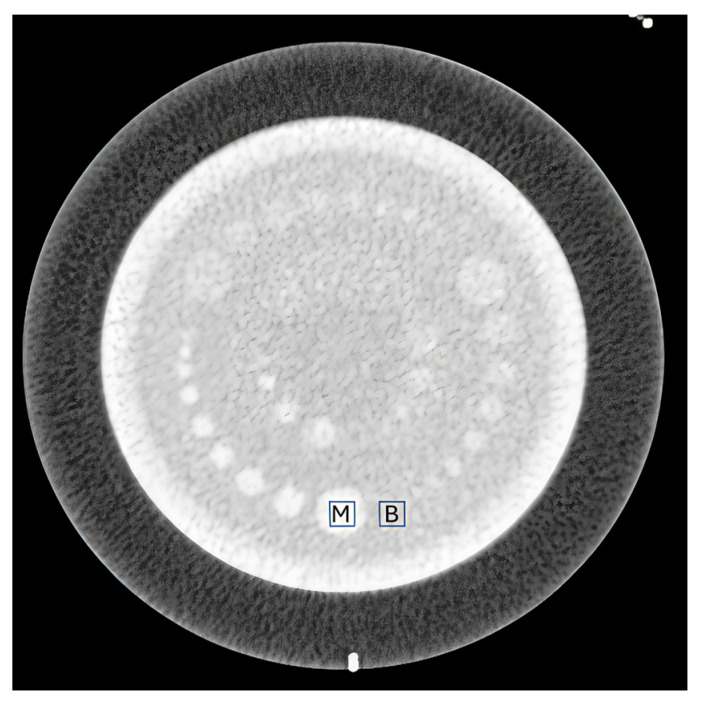
Measurement locations in the Catphan phantom. ROIs were placed in the low-contrast module: “M” indicates the measurement rod and “B” indicates the adjacent homogeneous background.

For quantitative measurements, circular regions of interest (ROIs; diameter, 10 mm) were placed within uniform background areas of the low-contrast module to measure SD and mean CT values, as shown in Figure 1.

Image noise was calculated as described in Equation (1).

The tube current was varied to simulate multiple dose conditions across all scanners.

All phantom scans were performed with the automatic exposure control (AEC) activated to evaluate each scanner’s dose-modulation behavior under clinical-like conditions.

Although many phantom studies recommend disabling AEC to standardize tube output, our study specifically aimed to assess how AEC performance varied among scanners under identical target SD settings.

This approach enabled comparison of vendor-specific AEC algorithms and their influence on dose–image-quality relationships in a manner that closely reflects real-world clinical operation.

The range of target SD values for AEC was set from 1 to 10, and corresponding images were acquired after a short warm-up period to stabilize tube output.

The lower limit for the radiation dose was defined as a volume CT dose index (CTDIvol) of 10 mGy.

(1) $$SD\;=\;\sqrt{\left\{{\left(SD_{M}\right)}^{2}+\;{\left(SD_{B}\right)}^{2}\right\}}$$

The CNR was calculated as displayed in Equation (2).

(2) $$CNR\;=\frac{\left(ROI_{M}-\;ROI_{B}\right)}{SD\_B}$$

Phantom measurements were taken three times for each condition, and the average values were used for analysis.

### 2.4. Image Quality Assessment

**Figure 2:**
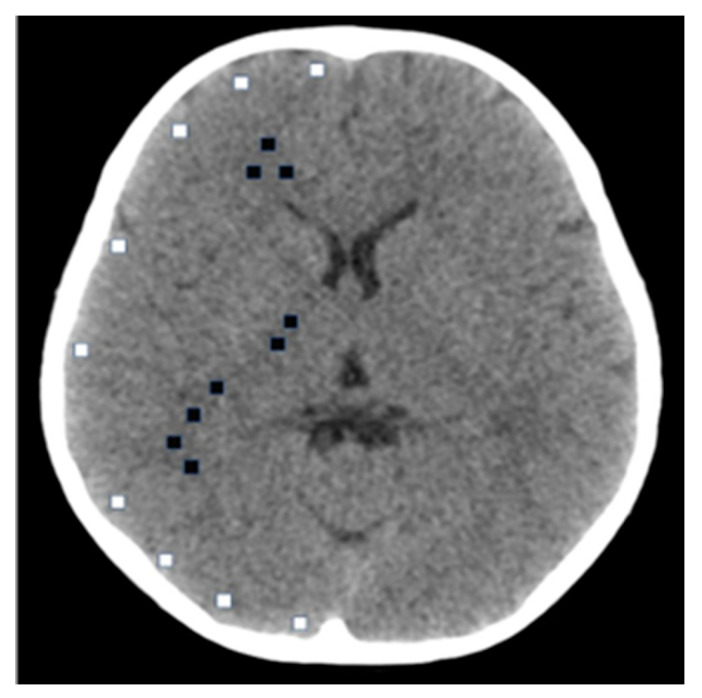
ROI placements for quantitative analysis of image quality in pediatric head CT. Multiple circular ROIs (10 × 10 pixels) were placed in the gray and white matter regions at the level of the basal ganglia. The mean CT values and standard deviations across all ROIs were used to calculate the SD and contrast-to-noise ratio (CNR) according to Equations (3) and (4).

For the clinical images, quantitative assessment was performed at the level of the basal ganglia, which provides a consistent representation of both gray and white matter (Figure 2).

Circular regions of interest (ROIs; 10 × 10 pixels) were symmetrically placed in the caudate nucleus (gray matter) and the adjacent internal capsule (white matter).

All ROI placements were performed by a single radiological technologist to ensure consistency.

Mean CT values and standard deviations were obtained from multiple ROIs placed in representative gray and white matter regions. For each dataset, the average of all gray matter ROIs and all white matter ROIs was used to calculate the contrast-to-noise ratio (CNR) and standard deviation (SD) in Equations (3) and (4).

Image noise was calculated by combining the SDs measured in gray and white matter using a root-sum-square approach, as given in Equation (3):

(3) $$SD=\surd {\left\{(SD\_GM\right)}^{2}+{\left(SD\_WM\right)}^{2}\}$$

The CNR was calculated as demonstrated in Equation (4).

(4) $$CNR=\frac{ROI_{GM}-ROI_{WM}}{SD_{WM}}$$

where the SD of the gray matter and the SD of the white matter represent the SD of the CT values in each region.

To account for uncertainty propagation, the variability of SD and CNR values was evaluated based on the standard error derived from the ROI distributions. This ensured consistency in quantitative assessment across all datasets.

Figure 2 shows the ROI placement in clinical images at the basal ganglia level. ROIs (10 × 10 pixels) were symmetrically placed in the caudate nucleus (gray matter) and the adjacent internal capsule (white matter).

### 2.5. Statistical Analysis

Image analysis was performed using CT-Measure software (version 0.96a; Japanese Society of Computed Tomography Technology [JSCT], Tokyo, Japan), the official integrated software for CT image measurements provided by JSCT. [20], and statistical analyses were conducted using R software (version 4.1.3; R Foundation for Statistical Computing, Vienna, Austria).

Dose–response relationships among CTDIvol, SD, and CNR were analyzed separately for each manufacturer using nonlinear regression models.

Regression curves were visually inspected to evaluate the relationships between radiation dose and image noise or contrast-to-noise ratio.

Facility-wise differences were minimal, as indicated by overlapping regression trends across sites.

The figure of merit (FOM) was calculated using Equation (5):

(5) $$FOM=\frac{A\times CNR^{B}}{CTDIvol}$$

The relationship between image noise (SD) and radiation dose (CTDIvol) was modeled using a power-law function:

(6) $$SD=A\times CTDI_{vol}^{-B}$$

Parameters A and B were obtained by regression fitting for each scanner and represent empirical constants describing the dose–image-quality relationship.

These coefficients were then used in Equation (5) to calculate the FOM for each manufacturer. A higher FOM indicates improved image quality per unit dose.

All analyses were performed in R, and the results are presented as regression trends and mean values for each manufacturer.

No inferential statistical tests were applied, as the analysis focused on dose-dependent tendencies rather than hypothesis testing.

The regression analyses were based on the power-law model described in Equation (6).

Each plot represents the relationship between CTDIvol and image noise (SD), contrast-to-noise ratio (CNR), or figure of merit (FOM), fitted separately for each manufacturer using nonlinear regression. Regression curves were generated to visualize scanner-dependent dose–image-quality characteristics.

### 2.6. Data Availability

The clinical and phantom imaging data used in this study cannot be shared publicly because they were obtained from collaborating medical institutions under data-use agreements that prohibit external distribution. These restrictions are in place to protect patient privacy and to comply with institutional research ethics policies.

Additionally, the R scripts developed for the statistical analysis contain institution-specific processing routines and are therefore not publicly available.

However, all analytical methods and procedures are fully described in the Section 2 to ensure transparency and reproducibility of the results.

## 3. Results

### 3.1. CT Values

In the phantom data, CT values remained stable across all dose settings and scanners.

The inter-facility differences were within ±4 Hounsfield units (HU), indicating consistent calibration and linearity across scanners. The 95% confidence intervals of mean CT values for each facility largely overlapped, suggesting high cross-scanner consistency. In contrast, the clinical CT values ranged from 30 to 40 HU and showed a slight decreasing trend as the radiation dose increased. This minor decrease likely reflects beam-hardening and reconstruction-kernel characteristics under higher tube-current conditions.

Table 3a,b summarize the pediatric head CT imaging conditions and corresponding mean CT values at each facility.

### 3.2. Noise (SD) Values

In both the phantom and clinical datasets, SD values decreased as radiation dose increased, showing a clear inverse relationship between dose and image noise.

**Figure 3:**
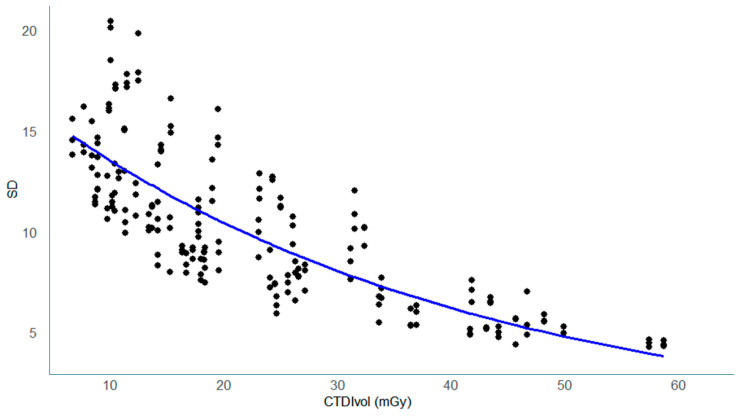
Dose dependence of image noise (SD) in the Catphan phantom. Scatter plot of image noise (SD) versus CTDIvol (mGy). Data points represent measurements from all facilities. The solid curve indicates the best-fit exponential decrease in SD with increasing dose.

**Figure 4:**
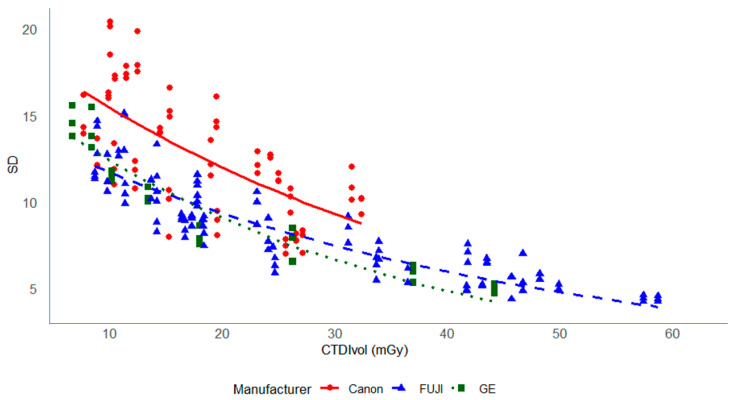
Dose dependence of image noise (SD) in the Catphan phantom by the manufacturer. This is similar to Figure 3, but the data points are categorized by manufacturer. Separate best-fit curves are shown for each manufacturer.

**Figure 5:**
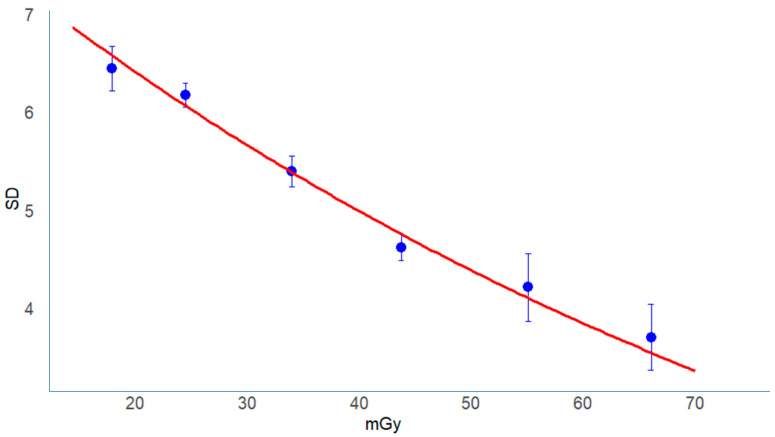
Dose dependence of image noise (SD) in clinical imaging. Scatter plot of image noise (SD) versus CTDIvol (mGy) in the clinical images. The best-fit curve demonstrates an exponential decrease in SD, with error bars indicating 95% confidence intervals.

The most pronounced reduction occurred between 10 and 20 mGy, after which the decrease gradually leveled off (Figure 3, Figure 4 and Figure 5).

In the phantom data, this trend was consistently observed across all facilities (Figure 3), indicating stable and dose-dependent noise behavior among scanners. When analyzed by manufacturer (Figure 4), similar patterns were observed for all systems, although FUJI scanners showed a slight plateau in SD above approximately 40 mGy, which may reflect differences in reconstruction kernel or iterative reconstruction strength.

In the clinical images (Figure 5), SD also decreased exponentially with increasing dose, following a pattern comparable to the phantom data.

This consistency between phantom and clinical results suggests that AEC-based noise control operated similarly across different scanners and protocols.

### 3.3. Relationship Between CNR and Dose

**Figure 6:**
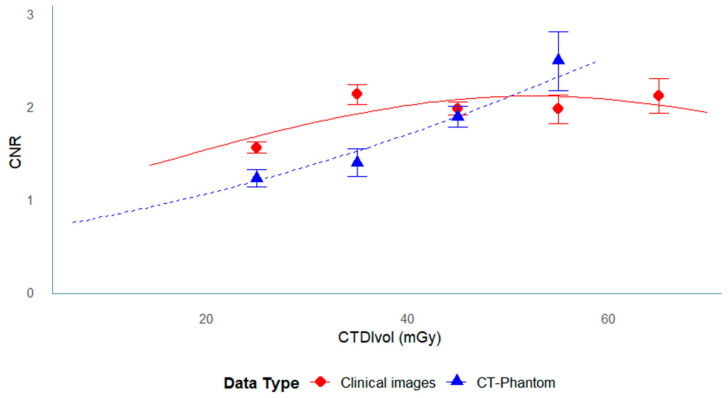
Dose dependence of CNR in the Catphan phantom. Scatter plot of CNR versus CTDIvol (mGy) in the phantom data. Data points represent all facilities, with the best-fit line showing proportional increase across the measured dose range.

**Figure 7:**
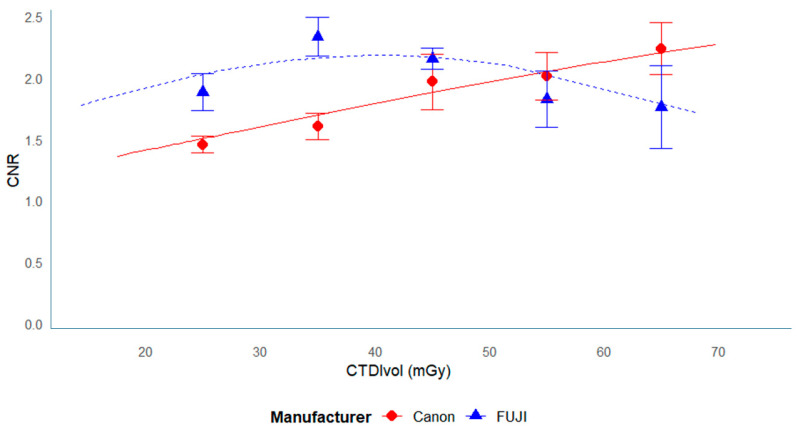
Dose dependence of CNR in clinical imaging. Scatter plot of CNR versus CTDIvol (mGy) in the clinical images. CNR increased with dose at lower exposure levels but reached a plateau above approximately 40 mGy.

The relationship between CNR and radiation dose is illustrated in Figure 6 and Figure 7.

In the phantom data (Figure 6), CNR increased with radiation dose throughout the measured range, showing a nearly proportional relationship up to approximately 40 mGy.

Beyond this range, the rate of increase gradually diminished, indicating a saturation tendency at higher doses.

In the clinical data (Figure 7), a similar pattern was observed—CNR increased with dose at lower exposure levels but reached a plateau above approximately 40 mGy.

This plateau was particularly evident in data from FUJI scanners, which may reflect differences in reconstruction kernels or iterative reconstruction algorithms rather than true dose limitation.

In this study, the interpretation of the optimal CNR level (≈2) was based on physical image-quality evaluation rather than observer performance.

A future reader-study or task-based detectability analysis is planned to validate whether this threshold corresponds to acceptable diagnostic performance in hemorrhage detection.

### 3.4. Relationship Between CNR and SD

**Figure 8:**
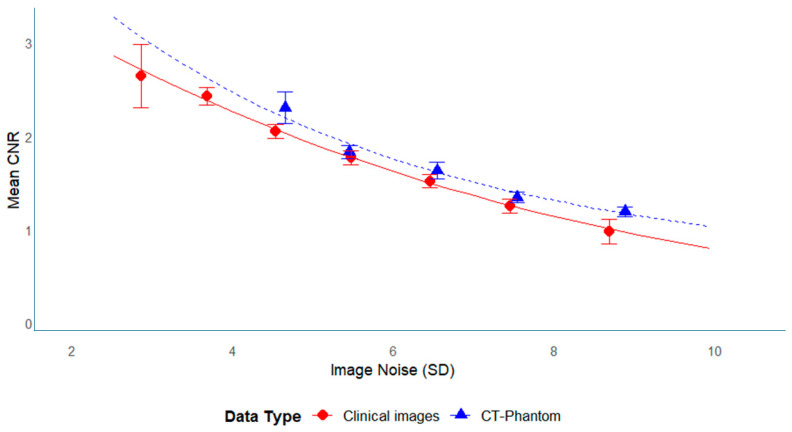
Association between image noise (SD) and CNR in clinical and phantom data. Scatter plot showing the relationship between image noise (SD) and CNR. The fitted curve illustrates the inverse correlation, with higher SD values corresponding to lower CNR values. Error bars indicate 95% confidence intervals.

Figure 8 illustrates the relationship between SD and CNR for both clinical and phantom data. In both datasets, CNR increased as SD decreased, demonstrating an inverse relationship between noise and the CNR. For the phantom data, this relationship followed an exponential trend (Equation (7)):

(7) $$CNR=exp\left(-0.002\cdot SD^{2}-0.145\cdot SD+1.43\right)$$

For the clinical data, a similar exponential trend was observed (Equation (8)):

(8) $$CNR\;=\;exp(0.0061\cdot {SD}^{2}\;-\;0.231\cdot SD\;+\;1.73)$$

Since CNR was defined as CNR = (CT_gray − CT_white)/SD_white, image noise (SD) was identified as the primary determinant of the CNR values. Table 4 summarizes the estimated CNR values at different SD levels, demonstrating that an SD of approximately 5 corresponds to a CNR of about 2. This consistency suggests that phantom-based measurements may apply to clinical settings.

### 3.5. FOM

#### 3.5.1. Statistical Equivalence Assessment

The distributions of the Figure of Merit (FOM) values were visually compared among the three manufacturers (Canon, FUJI, and GE).

The overall trends appeared similar, and no systematic differences in FOM were observed across systems. This indicates that image quality per unit dose was comparable among scanners from different vendors under the tested conditions.

#### 3.5.2. Modeling the Relationship Between FOM and CTDIvol

The relationship between FOM and CTDIvol for each manufacturer was approximated using a power function, as summarized below:

Canon: a = 6.25, b = −0.64

FUJI: a = 2.36, b = −0.26

GE data were limited to a narrow dose range and were therefore excluded from curve fitting. The negative exponents indicate that FOM decreased slightly as dose increased, reflecting diminishing gains in CNR improvement at higher exposure levels. Overall, similar power-law behavior was observed across manufacturers, suggesting that dose efficiency was maintained among systems.

## 4. Discussion

As most CT systems now incorporate automatic exposure control (AEC), selecting appropriate image-quality parameters remains challenging [7]. Lowering the upper limit of tube-current settings can effectively reduce dose. Future protocols should incorporate age and weight for further optimization [14]. This multicenter study investigated the relationship between dose and image quality in pediatric head CT scans, utilizing both clinical and phantom data. Our main findings were (1) CT values remained stable across facilities and systems, with inter-facility differences within ±4 HU; (2) image noise (SD) decreased as dose increased, especially between 10 and 20 mGy, and plateaued above 40 mGy; (3) CNR increased proportionally with dose in the phantom data but plateaued in the clinical data above approximately 40 mGy; (4) an inverse relationship between SD and CNR was observed, with phantom-based estimates aligning with clinical results; and (5) FOM values were not statistically significantly different among manufacturers, indicating a similar performance across CT systems.

Because the phantom evaluation followed the Japanese standard CT measurement methodology (JSRT), using uniform materials with consistent attenuation properties, the observed dose–image quality trends are considered technically reliable. Most facilities initially used protocols provided by the device manufacturer for pediatric applications. However, imaging conditions were inconsistent and even varied among facilities using equipment from the same manufacturer. Our clinical data showed a mean CTDIvol of 34.1 mGy (range: 14.4–69.8 mGy), which is below the 2020 Japanese DRL recommendation of 40 mGy for pediatric head CT (ages 1–5 years) [15]. Conversely, the mean CTDIvol reported in a U.S. community hospital was 27.3 mGy [16]. Although reductions in follow-up examinations and specific pediatric scenarios have been reported in the literature, the participating facilities in this study had not implemented formal follow-up dose-reduction schemes [21,22].

As shown in Figure 3, Figure 4 and Figure 5, the SD decreased curvilinearly with dose, consistent with the physical principle that image noise is inversely proportional to the square root of the tube current-time product (mAs):

(9) $$D\propto \frac{1}{\sqrt{mAs}}$$

This alignment with established physical laws strengthens the validity of our measurements [23]. Canon systems consistently displayed higher SD values compared to those of FUJI and GE, likely reflecting differences in AEC performance, target SD settings, or reconstruction algorithms. Canon’s lower target SD (2.5–3.0) suggests a strategy focused on reducing noise and potentially higher quality, whereas FUJI’s higher target SD (3.8–4.0) permits greater noise tolerance and dose reduction. A lower target SD (e.g., in Canon systems) generally reduces image noise and may improve the detection of subtle low-contrast lesions. However, proprietary reconstruction algorithms, differences in CT values, and device-specific noise characteristics contribute to variability in CNR [10].

CNR is an established and reliable metric for assessing image quality. This study suggests that an image noise level of SD ≈ 5 corresponds to a CNR of ≈2, which may serve as a practical benchmark for optimizing hemorrhage screening protocols in pediatric head CT. This threshold aligns with previous task-based detectability studies, indicating that a CNR in the range of 1.4–2 enables reliable detection of high-contrast abnormalities such as intracranial hemorrhage [11]. Future reader studies are planned to prospectively confirm whether these quantitative thresholds (SD ≈ 5 and CNR ≈ 2) correspond to acceptable diagnostic performance in pediatric TBI. In contrast, our proposed CNR target of 2 reflects the requirements for hemorrhage detection, a high-contrast abnormality, where higher diagnostic certainty is essential for pediatric TBI. Previous phantom-only investigations provided critical technical insights but did not address optimizing clinical imaging conditions [16]. By integrating multicenter clinical datasets with phantom measurements, we confirmed that dose–image quality relationships and thresholds (SD ≈ 5, CNR ≈ 2) are directly applicable to pediatric protocols. This clinical grounding sets our work apart from earlier studies and supports the use of phantom-based evaluation as a surrogate for clinical image quality assessment.

Our results also highlight the importance of optimizing protocols across manufacturers. CT values varied despite using the same phantom, likely reflecting differences in effective X-ray energy among vendors [24]. Clinical gray matter CT values ranged from 30 to 40 HU, consistent with previous reports indicating age-dependent decreases in attenuation [25]. These are attributed to both technical factors [26] and brain maturation [27]. These manufacturer-specific variations highlight the necessity of system-tailored optimization to achieve the desired balance between dose and diagnostic performance. Imaging conditions should be refined in conjunction with qualitative image review to ensure diagnostic accuracy [20]. Advanced techniques, such as deep learning image reconstruction (DLR), have demonstrated potential, with reports of 50% improvements in CNR [28], dose reductions of 46.4% in chest CT and 38.2% in abdominal CT [22], and pediatric head CT with CTDIvol as low as 12.5 mGy [29]. These innovations [30] may enhance protocol optimization further.

While SD and CNR were our primary outcomes, the Figure of Merit (FOM) offered a complementary dose-efficiency perspective [31]. In descriptive comparisons, we observed no systematic manufacturer-wise differences under the tested conditions, suggesting that the proposed SD/CNR targets are transferable if protocols are tuned to local AEC and reconstruction behavior. FOM offers a reproducible and system-tailored index that, when combined with physical measurements and qualitative image review by the authors, supports evidence-based standardization of pediatric head CT protocols.

For FUJI scanners, however, a decline rather than a plateau in CNR was observed above approximately 40 mGy. This may result from the stronger edge-preserving characteristics of FUJI’s iterative reconstruction kernel, which modify noise texture at high tube currents and can reduce the effective contrast between ROIs. The mean CNR in this study was 1.83 (range: 0.3–4.6), slightly lower than that reported in adults [32] and children [33]. Prior studies have demonstrated a saturation phenomenon, where excessive dose increases yield diminishing returns or even degrade image quality under certain conditions [22].

AEC was intentionally left active during phantom measurements to replicate actual clinical operation. Although this approach differs from guideline-based evaluation methods (e.g., ACR), it enhances clinical relevance by reflecting real-world scanning conditions. Technological advances in CT have enabled considerable dose reduction, but further improvements can be achieved through strategies such as low-voltage imaging [34] and minimizing the scanned range [35]. Radiologists and radiographers must be familiar with their systems and continue to optimize imaging parameters to reduce radiation exposure while maintaining diagnostic confidence.

Several limitations should be acknowledged. First, the phantom experiments were conducted with AEC enabled to replicate actual clinical operation; this differs from guideline-based methods (e.g., ACR), which typically disable AEC for standardized performance testing. While this approach enhances clinical relevance, it complicates direct comparison of intrinsic scanner performance. Second, only three manufacturers (Canon, FUJI, and GE) were included, limiting generalizability. Third, patient demographics such as sex, height, and weight were not collected for ethical reasons, preventing detailed assessment of body-size effects on dose modulation. Fourth, as a retrospective study, inter-observer variability in ROI placement was not assessed, and formal reader evaluation was not performed. Finally, spatial-resolution metrics such as the modulation transfer function (MTF) and task-based detectability indices were not included, though these would complement the current findings. In addition, hybrid iterative reconstruction (HIR) was not analyzed separately from filtered backprojection (FBP), because each participating facility applied the reconstruction method routinely used in its clinical workflow. The potential influence of reconstruction method differences on image noise and CNR is therefore recognized as a methodological limitation.

Additionally, although site-level variations were visually assessed, a mixed-effects model could not be applied because of the limited number of cases per facility. This constraint was acknowledged as a methodological limitation in the interpretation of manufacturer-based trends.

Although the CNR values derived from the Catphan phantom depend on the material composition and do not perfectly reproduce the X-ray attenuation of human tissues, the phantom-based analysis provided a controlled and quantitative framework for comparing scanner performance under standardized conditions.

These controlled measurements were subsequently validated using actual pediatric head CT data, demonstrating consistent dose–image-quality relationships across manufacturers and facilities.

This integrated approach—linking quantitative phantom assessment with multicenter clinical validation—represents the novelty and merit of this study.

The results indicate that phantom-derived dose–image-quality models can be used as a reference for protocol optimization in clinical practice, supporting safer pediatric head CT imaging with reduced radiation exposure while maintaining diagnostic confidence.

Future studies should include observer performance testing or reader studies to validate whether the proposed SD and CNR thresholds (SD ≈ 5, CNR ≈ 2) correspond to optimal diagnostic performance for hemorrhage detection. Prospective multicenter evaluations incorporating visual assessments, additional CT systems, and standardized protocols will further advance pediatric head CT optimization and harmonization.

The observed variability in CTDIvol (14.4–69.8 mGy) emphasizes the need for closer alignment of local practices with international reference levels. By combining phantom-based quantitative evaluation with emerging AI-driven and deep-learning reconstruction techniques, future efforts may enhance pediatric head CT protocol standardization and promote safer, evidence-based imaging worldwide. Harmonized, system-tailored protocols that integrate AEC behavior and modern reconstruction, including DLR and emerging transformer-based or AI-assisted approaches, are likely to further reduce radiation while maintaining diagnostic reliability [36,37].

## 5. Conclusions

Radiation doses in pediatric head CT varied considerably across facilities. We demonstrated that phantom-based measurements can be used to establish quantitative thresholds for image quality, with SD ≈ 5 and CNR ≈ 2 serving as practical optimization targets. These thresholds enable protocol standardization across different CT systems and support accurate hemorrhage detection in pediatric head trauma.

Furthermore, the wide inter-facility variation in CTDIvol highlights the need for greater harmonization of pediatric imaging protocols. Aligning local practice with international guidelines is essential to ensure both dose optimization and diagnostic reliability.

Future studies should require reading performance evaluations to confirm whether these quantitative thresholds correspond to optimal diagnostic accuracy in clinical practice.

By combining phantom-based quantitative evaluation with emerging techniques such as deep learning reconstruction, future efforts may advance pediatric head CT protocol standardization and promote safer, evidence-based imaging worldwide.

## Figures and Tables

**Table 1:** CT systems used at each participating facility.

Hospital Name	Manufacturer	Model Type	Detector Row Count	Sample Size
A	Canon (Toshiba) (a)	Aquilion PRIME	80	19
B	Canon (Toshiba)	Aquilion Prime SP	80	15
C	Canon (Toshiba)	Aquilion Prime SP	80	9
D	FUJI (HITACHI) (b)	Supria Grande	64	7
E	FUJI (HITACHI)	SCENARIA	64	16
F	FUJI (HITACHI)	Supria Optica	64	6
G	FUJI (HITACHI)	Supria	16	5
H	GE	Revolution EVO	256	13

Notes: (a) Toshiba models are listed under Canon, reflecting corporate rebranding. (b) HITACHI models are manufactured by FUJI. ample Size indicates the number of pediatric head CT examinations included for each scanner.

**Table 2:** (**a**) Scan parameters for pediatric head CT imaging at each facility. (**b**) AEC settings, reconstruction methods, and dose-reduction strategies.

(**a**)						
**Manufacturer**	**Hospital Name**	**Tube Voltage** **(kV)**	**Upper Tube Current (mA)**	**Shooting Time** **(s)**	**Slice Thickness** **(mm)**	**Helical Pitch**
Canon	A	120	250	0.5	5	0.637
Canon	B	120	350	0.5	5	0.637
Canon	C	120	270	0.6	5	0.637
FUJI	D	120	250	0.75	5	0.5781
FUJI	E	120	360	1	5	0.813
FUJI	F	120	200	0.8	5	0.5938
FUJI	G	120	180	1	5	0.8125
GE	H	120	520	0.5	5	0.984
(**b**)						
**Manufacturer**	**Hospital Name**	**Set SD**	**Helical Pitch**	**Image** **Reconstruction**		**Dose-Reduction Strategy**
Canon	A	2.5	0.637	None		Yes (reduced mA limit)
Canon	B	3	0.637	None		No
Canon	C	2.5	0.637	None		No
FUJI	D	4	0.5781	Smooth (level 3)		Yes (reduced SD target)
FUJI	E	3.8	0.813	Smooth (level 2)		No
FUJI	F	3.8	0.5938	None		No
FUJI	G	4	0.8125	None		No
GE	H	2.8	0.984	None		No

Note: Table 2a,b summarize the scanning conditions for pediatric head CT imaging at each facility. Table 2a lists the main acquisition parameters, whereas Table 2b provides details on AEC target SD, reconstruction algorithms, and the presence or absence of site-specific dose-reduction strategies. All examinations were performed under automatic exposure control (AEC). “None” indicates filtered backprojection (FBP), whereas “Smooth (level 2–3)” refers to hybrid iterative reconstruction (HIR). All scans were performed with automatic exposure control (AEC). Dose-reduction strategies represent site-specific approaches, such as reduced mA limit or target SD.

**Table 3:** (**a**) Phantom-based CTDIvol and CT values for each facility. Phantom-based measurements of CTDIvol and mean CT values obtained using the Catphan^®^ 600 under standardized scanning conditions. The phantom data represent scanner-specific output and physical performance independent of patient variability. (**b**) Clinical CTDIvol and CT values for each facility. Clinical CTDIvol and mean CT values derived from anonymized pediatric head CT examinations performed under routine protocols (ages 1–6 years). The clinical data reflect real-world dose levels and image intensities influenced by anatomical and protocol-specific variations.

(**a**)			
**Manufacturer**	**Hospital**	**CTDIvol (mGy) Mean (95% CI)**	**CT Value (HU) Mean (95% CI)**
Canon	A	17.08(11.43–22.73)	60.8(60.02–61.59)
	B	18.61(12.47–24.75)	61.54(60.98–62.10)
	C	19.5(13.91–25.09)	62.89(61.93–63.85)
FUJI	D	31.42(21.14–41.71)	60.63(59.92–61.55)
	E	28.28(15.05–41.5)	58.26(57.34–59.19)
	F	25.51(14.06–36.97)	59.35(57.77–60.94)
	G	26.13(16.41–35.85)	60.89(58.81–62.97)
GE	H	19.71(8.6–30.83)	59.75(58.31–61.19)
(**b**)			
**Manufacturer**	**Hospital**	**CTDIvol (mGy) Mean (95% CI)**	**CT Value (HU) Mean (95% CI)**
Canon	A	23.11 (21.68–24.54)	33.32 (32.61–34.04)
	B	37.78 (24.43–51.12)	35.12 (33.88–36.36)
	C	30.98 (25.54–36.41)	44.07 (40.59–47.55)
FUJI	D	26.86 (20.75–32.96)	39.55 (37.86–41.24)
	E	42.44 (38.71–46.18)	37.53 (36.59–38.47)
	F	30.78 (27.42–34.15)	39.63 (39.05–40.21)
	G	35.46 (19.72–51.2)	36.6 (35.43–37.76)
GE	H	42.18 (39.97–44.39)	32.95 (31.86–34.03)

Note: CI = confidence interval. The mean CT value represents the average of gray and white matter ROI measurements obtained from the Catphan phantom’s uniformity and contrast modules. The mean CT value represents the average of gray and white matter ROI measurements obtained from the caudate nucleus (gray matter) and internal capsule (white matter). The phantom and clinical datasets are presented separately (Table 3a,b) to allow direct comparison between controlled physical measurements and real-world pediatric CT imaging conditions.

**Table 4:** Estimated CNR values corresponding to different SD levels in clinical and phantom data based on exponential models (Equations (7) and (8)).

	CNR	
SD Value	Clinical Data	Phantom Data
1	3.612	4.512
2	3.106	3.648
3	2.658	2.985
4	2.266	2.473
5	1.923	2.074
6	1.625	1.76
7	1.367	1.513
8	1.145	1.316
9	0.955	1.158
10	0.793	1.033

## Data Availability

Clinical data are not publicly available due to patient privacy. Anonymized datasets and R scripts used for statistical analysis are available from the corresponding author upon reasonable request.

## Abbreviations

The following abbreviations are used in this manuscript:

Abbreviation	Definition
AEC	Automatic exposure control
CNR	Contrast-to-noise ratio
CTDIvol	Volume CT dose index
DLR	Deep learning reconstruction
DRL	Diagnostic reference level
FOM	Figure of merit
HU	Hounsfield unit
ROI	Region of interest
SD	Standard deviation
TBI	Traumatic brain injury
